# Fat-Soluble Vitamins in Standard vs. Liposomal Form Enriched with Vitamin K2 in Cystic Fibrosis: A Randomized Multi-Center Trial

**DOI:** 10.3390/jcm11020462

**Published:** 2022-01-17

**Authors:** Jan Krzysztof Nowak, Patrycja Krzyżanowska-Jankowska, Sławomira Drzymała-Czyż, Joanna Goździk-Spychalska, Irena Wojsyk-Banaszak, Wojciech Skorupa, Ewa Sapiejka, Anna Miśkiewicz-Chotnicka, Jan Brylak, Barbara Zielińska-Psuja, Aleksandra Lisowska, Jarosław Walkowiak

**Affiliations:** 1 Department of Pediatric Gastroenterology and Metabolic Diseases, Poznan University of Medical Sciences, Szpitalna 27/33, 60-572 Poznan, Poland; jan.nowak@ump.edu.pl (J.K.N.); pkrzyzanowska@ump.edu.pl (P.K.-J.); drzymala@ump.edu.pl (S.D.-C.); chotnicka@ump.edu.pl (A.M.-C.); jan.brylak@student.ump.edu.pl (J.B.); alisowska@ump.edu.pl (A.L.); 2 Department of Bromatology, Poznan University of Medical Sciences, Marcelinska 42, 60-354 Poznan, Poland; 3 Department of Pulmonology, Allergology and Respiratory Oncology, Poznan University of Medical Sciences, Szamarzewskiego 84, 60-569 Poznan, Poland; jogoz@ump.edu.pl; 4 Department of Pediatric Pneumonology, Allergology and Clinical Immunology, Poznan University of Medical Sciences, Szpitalna 27/33, 60-572 Poznan, Poland; iwojsyk@ump.edu.pl; 5 Department of Lung Diseases, Institute for Tuberculosis and Lung Diseases, Plocka 26, 01-138 Warsaw, Poland; w.skorupa@igichp.edu.pl; 6 The Specialist Centre for Medical Care of Mother and Child, Polanki 119, 80-308 Gdańsk, Poland; e.sapiejka@wp.pl; 7 Department of Toxicology, Poznan University of Medical Sciences, Dojazd 30, 60-631 Poznan, Poland; bzielin@ump.edu.pl

**Keywords:** cystic fibrosis, vitamin A, retinol, vitamin D, cholecalciferol, vitamin E, tocopherol, vitamin K, menaquinone, liposome

## Abstract

Background: We aimed to assess a liposomal fat-soluble vitamin formulation containing vitamin K2 with standard treatment in cystic fibrosis (CF). Methods: A multi-center randomized controlled trial was carried out in 100 pancreatic-insufficient patients with CF. The liposomal formulation contained vitamin A as retinyl palmitate (2667 IU daily) and beta-carotene (1333 IU), D3 (4000 IU), E (150 IU), K1 (2 mg), and K2 as menaquinone-7 (400 µg). It was compared with the standard vitamin preparations in the closest possible doses (2500 IU, 1428 IU, 4000 IU, 150 IU, 2.14 mg, respectively; no vitamin K2) over 3 months. Results: Forty-two patients finished the trial in the liposomal and 49 in the control group (overall 91 pts: 22.6 ± 7.6 years, 62.6% female, BMI 19.9 ± 2.8 kg/m^2^, FEV1% 70% ± 30%). The main outcome was the change of vitamin status in the serum during the study (liposomal vs. standard): all-trans-retinol (+1.48 ± 95.9 vs. −43.1 ± 121.4 ng/mL, *p* = 0.054), 25-hydroxyvitamin D3 (+9.7 ± 13.4 vs. +2.0 ± 9.8 ng/mL, *p* = 0.004), α-tocopherol (+1.5 ± 2.5 vs. −0.2 ± 1.6 µg/mL, *p* < 0.001), %undercarboxylated osteocalcin (−17.2 ± 24.8% vs. −8.3 ± 18.5%, *p* = 0.061). The secondary outcome was the vitamin status at the trial end: all-trans-retinol (370.0 ± 116.5 vs. 323.1 ± 100.6 ng/mL, *p* = 0.045), 25-hydroxyvitamin D3 (43.2 ± 16.6 vs. 32.7 ± 11.5 ng/mL, *p* < 0.001), α-tocopherol (9.0 ± 3.1 vs. 7.7 ± 3.0 µg/mL, *p* = 0.037), %undercarboxylated osteocalcin (13.0 ± 11.2% vs. 22.7 ± 22.0%, *p* = 0.008). Conclusion: The liposomal fat-soluble vitamin supplement containing vitamin K2 was superior to the standard form in delivering vitamin D3 and E in pancreatic-insufficient patients with CF. The supplement was also more effective in strengthening vitamin K-dependent carboxylation, and could improve vitamin A status.

## 1. Introduction

Deficiencies of fat-soluble vitamins remain a current therapeutic challenge in cystic fibrosis (CF). A large share of the patients exhibit vitamin K or D deficiencies, and the prevalence of vitamin E and A insufficiencies is also considerable. In many cases, increases of vitamin doses are not efficacious, and intractable CF-related fat-soluble vitamin deficiencies are diagnosed, especially with regard to vitamin K [1,2]. Additionally, for this reason, the current ESPEN-ESPGHAN-ECFS guidelines for fat-soluble vitamin supplementation [3] leave many options of therapy individualization and are rather cautious towards supplementation of vitamin A, which has the highest potential for toxicity at overdose.

The response to the problem of deficiencies is usually focused on providing complex fat-soluble vitamin supplementation [4] with the necessary personalization through add-on treatment. In most cases, however, standard medium chain triglyceride-based formulations are used. The liposomal form is available only for vitamin E (TPGS: d-α-tocopherol polyethylene glycol 1000 succinate) within complex supplements [4]. There are also additional forms of fat-soluble vitamins not routinely used in CF, for example, 25-hydroxyvitamin D or specific carotenoids.

One of the alternative forms of fat-soluble vitamin K is menaquinone-7 (MK-7), which belongs to compounds classified as vitamin K2, and is considered a novel food under the EU law. MK-7 is known to be naturally produced by the intestinal microbiota and to have a longer half-time in the blood compared with phylloquinone [5], the standard vitamin K1 used in CF. In postmenopausal women, MK-7 is able to reduce risk fracture, testifying of its biological activity [6]. In CF, however, MK-7 or any other form of vitamin K2 has never been explored.

Apart from using new forms of vitamins to address deficiency, we may also employ innovative delivery technologies. Examples of using new delivery methods in medicine come from other fields, for example, amphotericin B has a liposomal formulation and voriconazole can be administered as a cyclodextrin complex. However, in CF, few methods of fat-soluble vitamin delivery were researched apart from TPGS and an organized lipid matrix [7].

In this study, we focused on a novel liposomal formulation of vitamins A, D, E, and K, which was enriched with vitamin K2 (Cystisorb Liposomal, Norsa Pharma, Cracov, Poland). The main purpose of the preparation is to address the problem of intractable fat-soluble vitamin deficiencies in pancreatic-insufficient CF patients. This study’s primary aim was to compare a novel liposomal formulation of fat-soluble vitamins enriched with vitamin K2 with vitamins in their standard forms.

## 2. Materials and Methods

This was a randomized open-label multi-center trial. The eligibility criteria were CF with exocrine pancreatic insufficiency and an age of 12–55 years. Patients with liver cirrhosis [8], pregnancy, and expected lung transplantation were excluded from the study. The trial was conducted in the hospitals of Poznan University of Medical Sciences (Poznan, Poland), the National Institute for Tuberculosis (Warsaw, Poland), and the Gdansk CF Outpatient’s Clinic (Gdansk, Poland) in the year 2019.

Table 1 summarizes the information on vitamin doses in the trial. The investigated intervention was a novel liposomal formulation of fat-soluble vitamins (Cystisorb Liposomal, Norsa Pharma, Cracov, Poland): vitamin A (800 RAE as retinyl palmitate/day, 200 RAE as beta-carotene/day), vitamin D3 (4000 IU as cholecalciferol/day), vitamin E 150 IU/day (including TPGS: d-α-tocopheryl polyethylene glycol 1000 succinate, as well as alpha-tocopherol), vitamin K1 and K2 (2 mg of phylloquinone/day and 0.4 mg of menaquinone-7/day), for 90 days. In the control group, standard formulations of fat-soluble vitamins were provided: vitamin A (750 RAE as retinyl palmitate/day; beta-carotene average 214 RAE/day), vitamin D3 (4000 IU as cholecalciferol/day), vitamin E (150 IU as alpha-tocopherol/day), and vitamin K1 (2.14 mg of phylloquinone/day) for 90 days as well. In the control group, beta-carotene was given once per week, vitamin E dosage was 1 or 2 capsules every other day, and vitamin K1 was given thrice a week. This dosing scheme was used to achieve the daily dose most closely matching the active treatment arm. The patients were advised to take the supplements with meals.

**Figure 1:**
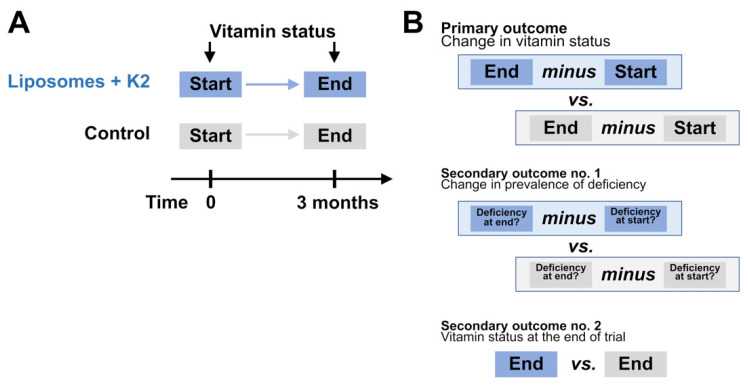
A simplified scheme of the study (**A**) illustrating the primary and secondary outcomes (**B**).

The primary outcome was the change from the baseline in vitamin status in the serum: vitamin A (all-trans-retinol), vitamin D3 (25-hydroxyvitamin D), vitamin E (alpha-tocopherol), vitamin K (% of uncarboxylated osteocalcin—deficiency indicated by high level) after 90 days (i.e., blood draw at the end of the study, within the last 10 days of the intervention). The secondary outcomes were: (1) the change from the baseline in the prevalence of vitamin A, D, E, and K deficiency and (2) the values of the above indicators (A, D, E, K, and also as deficiency prevalence) at the end of the study. The outcomes are illustrated in Figure 1.

The assessment of the serum concentrations of all-trans-retinol and α-tocopherol (as well as the additional control assessments of β-carotene) was conducted using high-performance liquid chromatography (Hewlett Packard 1100 HPLC, Agilent, Waldbronn, Germany; Santa Clara, CA, USA) [9,10,11]. The concentrations of 25-hydroxyvitamin D3 were measured with chemiluminescence on the Architect (Abbott, Chicago, IL, USA). The efficacy of vitamin K-dependent carboxylation was measured with a biomarker: the percentage of undercarboxylated osteocalcin, derived from the measurements of both carboxylated and undercarboxylated osteocalcin using ELISA (Gla-type Osteocalcin EIA and Glu-type Osteocalcin EIA (Takara Bio Inc., Otsu, Japan). After sampling, the blood was protected from light, centrifuged in cooling conditions and stored at −80 °C. Sample transport occurred in dry ice only. Until the assessment, the samples were also stored in −80 °C. The measurements in all the samples were conducted within a period of a few days to minimalize errors. Adequate vitamin levels were defined as ≥300 ng/mL of all-trans-retinol, ≥20 ng/mL of 25-hydroxyvitamin D3, ≥5 µg/mL of alpha-tocopherol, and ≤20% of ucOC.

The sample size was determined assuming the power of 85%, the size of the observed effect equaling two-thirds of the standard deviation of the measured parameters, and the significance value 0.05. G*Power 3.1 (University of Dusseldorf, Dusseldorf, Germany) was used for the calculations. Additionally, we assumed that up to 20% of patients might be lost to follow-up, and thus we obtained the planned sample size of 100. It should be added that the sample size was calculated with the primary outcome in mind. The additional prevalence analysis (a secondary outcome) is underpowered and meant to provide a general overview of deficiency prevalence through binary classification of vitamin status. This inevitably leads to loss of information, and therefore, the focus should be on the primary outcome.

The random allocation sequence was generated using a computer algorithm. Block randomization was conducted depending on the center, sex, and nutritional status (above or below BMI 21 kg/m^2^). The block size of four was chosen due to the number of compensated confounding factors. The planned allocation ratio was 1:1. No interim analyses were performed, and no stopping guidelines were set.

At recruitment, the patient received an individual code from the physician. 

The random allocation sequence was generated using a computer algorithm. Block randomization was conducted using three variables. The first was the center, since each center had its own randomization list. All these individual lists were managed by one coordinator located in Poznan. Each list allowed for randomization depending on sex and nutritional status (above or below BMI 21 kg/m^2^). The stratification threshold of 21 kg/m^2^ was selected because it was the median body mass from our previous studies in the Polish population of pancreatic-insufficient patients with CF. The block size of four was chosen due to the number of compensated confounding factors. A larger block size was not used to prevent the lack of balance in clinical characteristics between the groups. The planned allocation ratio was 1:1. No interim analyses were performed because the blood was to be sampled only at the end of the study and assayed in one batch. No stopping guidelines were set, but cautious vigilance on the part of treating physicians has been encouraged, and all the patients received direct contact to a physician in Poznan, who immediately reacted to any reported side effects and discussed them with the study team. To maximize patient safety, the presence of side-effects thought to be related to taking the supplement resulted in exclusion from the study. Of note, in a previous trial, a liposomal formulation of vitamins A, D, E, and K was tested in 28 patients over three months, and no side effects have been reported [12].

At recruitment, the physician categorized the patient as male/female and above or below the BMI of 21 kg/m^2^. He accessed a list prepared for this category in the given center and read the next available code from the list. Thus, the code was taken sequentially from a list specific for a center, sex, and nutritional status. After having received the unique code, the patient contacted the coordinating center in Poznan, providing the code and the preferred address for vitamin shipping. According to group allocation (pre-defined for the unique code), liposomal or standard vitamins were then shipped. The patients received two more parcels—one and two months later—which contained the doses of vitamins necessary to continue the study. This was done in order to ensure adequate storage conditions. The patients kept the liposomal formulation in the refrigerator. A control visit was planned within a few days at the end of the study period. The patients were compensated for their participation in the trial with complex fat-soluble vitamin supplements. The random allocation sequence was generated by a researcher in the coordinating center. The patients were recruited by their CF physicians. The participants were assigned to interventions by a researcher in the coordinating center. The investigated vitamin supplements differed, and the patient could discern whether the allocation was to the intervention or the control group.

Comparisons between the groups were performed using the unequal variances *t*-test (the Welch test) for continuous variables and the Fisher’s exact test where applicable. Two-sided p values are reported. Statistica 12 (TIBCO, Palo Alto, CA, USA) and R 3.6.0 (R Foundation, Vienna, Austria) with a ggpubr data visualization package were used to perform analysis and prepare plots, respectively.

The study was approved by the bioethical committee at Poznan University of Medical Sciences (417/17 with the associated amendments). The research adhered to the tenets of the Declaration of Helsinki. All the participants and/or their parents/guardians provided informed written consent. The study was registered in the German Clinical Trials Registry under the number DRKS00018814.

## 3. Results

Overall, 100 pancreatic insufficient CF patients were enrolled, of whom 91 finished the study. In the intervention group, three participants withdrew because of gastric discomfort, two patients did not contact the coordinating center, and one missed the follow-up visit. In the control group, two patients withdrew after receiving parcels and discovering that they were allocated to standard vitamins, and one did not contact the coordinating center. All the patients for whom the data at follow-up were available finished the study by adhering to the intervention or control protocol. Therefore, the data for all these patients were included in the analyses.

All the patients were enrolled in 2019 and the follow-up visits also took place in 2019. The trial ended as predicted after all the patients finished follow-up. 

**Figure 2:**
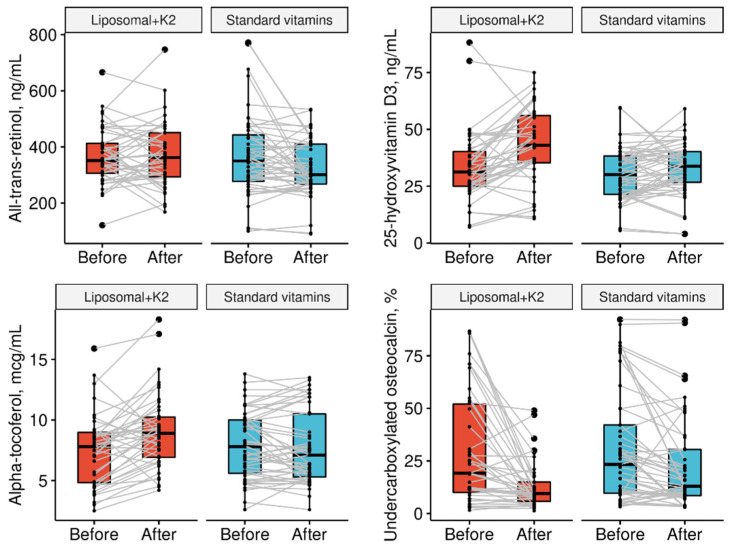
Vitamin A, D, E, and K status in pancreatic-insufficient CF patients before and after supplementation with the novel liposomal formulation or standard forms.

The baseline demographic and clinical characteristics of the groups are presented in Table 2. The doses of fat-soluble vitamins prior to the study are summarized in Table 3. The changes in vitamin concentrations (the primary outcome) and their levels at the end of the trial (a secondary outcome) are presented in Table 4 and Figure 2. The changes in deficiency prevalence are shown in Table 5.

## 4. Discussion

This is the first study to investigate a formulation including all fat-soluble vitamins in the liposomal form in CF. The study was conducted in pancreatic-insufficient patients not taking CFTR potentiators/correctors. We demonstrated that the supplementation of vitamin D in liposomes was more efficient compared with the standard supplements. Moreover, we found evidence of improved bioavailability of vitamin E and A in the liposomal form. Finally, compound administration of liposomal vitamins K1 and K2 can significantly ameliorate the functional vitamin K status.

### 4.1. Studied Group

The study group was mostly comprised of young CF patients with moderate to severe disease, all of whom had exocrine pancreatic insufficiency. Most of the patients were female, as women were more willing to participate in research. The average nutritional status was acceptable but not optimal. High rates of colonization with Pseudomonas aeruginosa may reflect not only a severe disease course, but also relate to past limitations in home CF care. Stratified randomization succeeded at keeping the sex ratio and nutritional status similar in both studied subgroups. Yet, despite the lack of statistically significant differences, it would seem that in some aspects, the liposomal group had a more severe disease course (FEV1%, GERD, *P. aeruginosa*, baseline vitamin doses), possibly slightly reducing the observed superiority of liposomes. None of the patients used CFTR potentiators/correctors as they were not available to patients in Poland at the time of the study because of the high cost. By contrast, all the patients used dornase alpha, most received short-acting beta-agonists, ursodeoxycholic acid, and inhalations of saline, and half took inhaled steroids, long-acting beta-agonists, and expectorants. All patients but one supplemented pancreatic enzymes (a case of enzyme intolerance: adequate nutritional status despite mild steatorrhea). In summary, this group included adolescents and young adults with moderate to severe CF who are frequent visitors of CF outpatients’ clinics and who might require additional attention, also regarding fat-soluble vitamin supplementation.

### 4.2. Vitamin A

The dose of vitamin A was minimally higher (3%) in the investigated group and had a slightly different retinol/beta-carotene balance. These changes were necessary because of practicalities and should not have influenced the results. It should also be considered that mean doses of vitamin A prior to the study were 1937 RAE vs. 1382 RAE in the control group, which could have made it more difficult to demonstrate a beneficial effect of the liposomal formulation. The final vitamin A concentration in the liposomal group was 14% higher compared with the control, suggesting a beneficial effect of the liposomal formulation.

### 4.3. Vitamin D3

The effect of the liposomal formulation on vitamin D bioavailability was important, as evidenced by high levels at the end of the trial. Consequently, one could even envisage that a dedicated liposomal vitamin D supplement could be devised for patients who require a targeted treatment of intractable deficiency. This could be further trialed even in liver cirrhosis, where intrinsic vitamin D metabolism is disturbed. It could be that the high bioavailability of liposomal vitamin D would exploit the remaining reserves of hepatic vitamin D hydroxylation. 

Only 8% of patients in the control group exhibited vitamin D deficiency. The liposomal form could prove useful for maintaining higher levels of vitamin D, if this was considered beneficial. In fact, optimal vitamin D levels remain the subject of debate despite intense research (even when healthy adults alone are considered).

### 4.4. Vitamin E

Mean daily doses of vitamin E before the trial were relatively high in the study group (290 IU vs. 241 IU in the control group). At baseline, the frequency of vitamin E deficiency was over 50% greater in the liposomal group as well. The fourfold reduction in vitamin E deficiency in the study group did not prove statistically significant. However, both vitamin E status change and final vitamin E concentration were more beneficial in the liposomal (TPGS) group. Of note, vitamin E was formulated not as 100% TPGS, but with the addition of tocopherol. Nevertheless, these results provide the much-needed rationale for the widespread use of TPGS in pancreatic insufficient CF patients.

### 4.5. Vitamin K

The compound supplementation with liposomal vitamin K1 and K2 as MK-7 yielded promising results. Both the final prevalence of insufficiency and the final undercarboxylated osteocalcin were positively influenced. Because of the study design, it is impossible to determine, on the basis of the obtained results, whether it was the liposomal form of vitamin K1 or the addition of vitamin K2 that proved decisive. The dose of vitamin K2 was higher than typically used in studies of osteoporosis in postmenopausal women, as needed because of malabsorption. MK-7 possesses high carboxylation activity and a longer half-life compared with vitamin K1. Taking into account results of our previous research on liposomal and cyclodextrin forms of fat-soluble vitamins (in review), it seems that both the standard vitamin K1 dose and a considerable dose of vitamin K2 were necessary to obtain the observed effects. 

### 4.6. Safety

No serious adverse effects have been linked to any of the studied formulations during the trial. The gastric discomfort reported by three patients taking the novel liposomal supplement started several days after inclusion. The discomfort recurred after supplement ingestion and resolved spontaneously after the supplement was stopped. Although the symptom was mild, it was advised that the patients withdraw from the study. Because the remaining patients did not report gastric symptoms despite supplement use over a period of three months, it may be concluded that gastric discomfort might develop in less than 1 in 10 patients, and if it does, the effect is seen early. 

### 4.7. Additional Consideration for the Interpretation of Trial Results

The value of this study is not limited to the comparison of the liposomal formulation with the standard form. In fact, all the patients in the control arm received the same doses of fat-soluble vitamins in a three-month period. This highlighted the need for close monitoring of vitamin A levels, risk for vitamin K deficiency despite systematic supplementation, and the potential to obtain non-deficient vitamin D levels even with 4000 IU of a standard supplement. These findings may be useful for setting guidelines on fat-soluble vitamin supplementation in the future [3]. 

The limitations of this research do not undermine the main conclusions. Firstly, the study was performed in Poland, where CF care has greatly improved in the past two decades, but is still not on par with Western countries [13]. This enabled the study to be performed in a population not using CFTR modulators/potentiators. Secondly, in the control group, vitamin E, beta-carotene, and vitamin K were given using a weekly schedule, but not daily. The doses and the composition of vitamin A supplementation slightly differed. Overall, the study was designed to provide the best comparison practically possible. Third, the few patients who withdrew from the study did not come for the follow-up visits, precluding intention-to-treat analysis. Fourth, vitamin K status was measured using osteocalcin undercarboxylation, which is not the only, but arguably the most widely accepted biomarker of vitamin K deficiency. Fifth, while in the liposomal group, patients took only one dose per day, in the control group they took, on average, four pills/capsules daily. If the patients were more likely to take the liposomal formulation more systematically because of an easier dosing regimen, this may also present an additional benefit of the liposomal formulation under real-life conditions of product use. Yet, because the patients were used to taking vitamins regularly, there seems to be little risk of bias in this aspect. It should also be underscored that patients with liver cirrhosis were not recruited for the study. A relatively large percentage of CF patients with liver disease results from the use of highly inclusive criteria [14]. The dietary patterns and pancreatic enzyme dosing were consistent during the study.

The study may be generalized to pancreatic-insufficient patients with CF not receiving CFTR modulator/potentiator therapy. However, it should also be stressed that the influence of these modern therapies on fat-soluble vitamin status has not been explored and it is uncertain in patients who have already developed exocrine pancreatic insufficiency. Moreover, fat-soluble vitamin deficiencies may occur in patients with malabsorption caused by diseases other than CF.

### 4.8. Context in the Literature

TPGS is the most widely adopted innovation in the supplementation of fat-soluble vitamins to patients with CF. Its beneficial pharmacokinetic properties have been demonstrated for γ-tocopherol, and it probably impacts the bioavailability of other tocopherols as well [4]. Another useful solution in this area includes multivitamin supplements dedicated to CF patients, which have been available (from various suppliers) for over a decade and are widely taken, often despite considerable costs [15]. The beneficial effects of one such supplement were observed in a nonrandomized trial (before–after design) involving 14 patients with CF, where an increase in the serum concentration of supplemented compounds was observed [16]. The product investigated in the aforementioned study included additional antioxidants, in line with current trends [15,16].

### 4.9. The Role of Fat-Soluble Vitamins in Cystic Fibrosis

Several factors contribute to the development of fat-soluble deficiencies in patients with CF. Digestion and absorption of fats is impaired in the majority of patients. This typically correlates with pancreatic insufficiency, but may also be related to liver disease, disturbance of primary biliary acid synthesis, as well as altered enterohepatic circulation and inadequate bile release from the gallbladder [17,18]. Patients with CF also have an increased requirement for fat-soluble vitamins because of ongoing inflammation and oxidative stress. In a fraction of patients, bowel resection contributes to fat-soluble vitamin deficiencies as well. Finally, in practice, it is not uncommon that poor adherence hinders long-term efforts to normalize fat-soluble vitamin levels [19]. 

In our experience, patients often justify poor adherence by organizational and financial burdens. Indeed, even if the need to supplement vitamins is widely understood in the CF community because of the awareness of the risk of severe symptoms, difficulties encountered every day by the patients may prevent full adherence. In the past, deficiencies led to night blindness and problems with blood coagulation. Such cases are still reported even today, reminding us of the important role of fat-soluble vitamin supplementation in CF [20,21]. The results of our study may indicate that the scale of self-declared supplementation of fat-soluble vitamins in some cases is exaggerated. This is best seen when vitamin K1 supplementation is analyzed—although high daily doses were reported before the trial, the uniform smaller dose given during the study was not associated with increases in osteocalcin undercarboxylation (i.e., worse vitamin K status). On the contrary, there seemed to be a beneficial trend, and the prevalence of vitamin K deficiency in the control group was cut by a third during the trial, despite these seemingly smaller doses of phytomenadione. An important consideration for vitamin K deficiency is antibiotic therapy. However, intractable vitamin K deficiency is difficult to predict, and does not seem to result from easily identifiable characteristics of CF [1,8].

## 5. Conclusions

In conclusion, the study demonstrated that a novel liposomal fat-soluble vitamin supplement enriched with vitamin K2 is superior to the standard fat-soluble vitamin supplements with regard to all supplemented vitamins, especially D and K.

## Figures and Tables

**Table 1:** Mean daily doses of fat-soluble vitamins in the intervention and the control group. The dose of vitamin A was 3.7% larger in the intervention group. The vitamin K1 dose was 7% larger in the control group.

Vitamin	Intervention (Liposomal + K2)	Control (Standard)
Vitamin A—retinyl palmitate	800 RAE	750 RAE
Vitamin A—β-carotene	200 RAE	214 RAE
Vitamin A, sum	1000 RAE	964 RAE
Vitamin D3—cholecalciferol	4000 IU	4000 IU
Vitamin E—α-tocoferol	150 IU inlc. TPGS	150 IU
Vitamin K1	2 mg	2.14 mg
Vitamin K2—menaquinone-7	400 µg	

**Table 2:** Baseline characteristics of patients included in the trial. Mean ± SD or percentage are provided.

Parameter	All Patients *N* = 91	Liposomal *N* = 42	Standard Vitamins *N* = 49	*p*
Age, years	22.6 ± 7.6 20.9 [16.5–26.8]	23.5 ± 7.4 21.5 [17.4–28.0]	21.9 ± 7.8 19.3 [15.9–24.0]	0.301
Sex, % women	62.6%	59.5%	65.3%	0.665
Mass, kg	54.2 ± 11.8 52.0 [46.0–60.0]	55.0 ± 11.5 55.5 [46.0–62.0]	53.4 ± 12.1 50.0 [45.5–60.0]	0.513
Height, cm	164.3 ± 9.3 165.0 [157.0-173.0]	166.1 ± 9.5 168.0 [161.0–173.0]	162.9 ± 8.9 162.0 [157.0–168.0]	0.102
BMI, kg/m^2^	19.9 ± 2.8 19.4 [18.0–21.5]	19.8 ± 2.8 19.6 [18.0–21.05]	19.9 ± 2.9 19.1 [18.0–21.55]	0.848
FEV1%	70.1 ± 30.3 76.0 [42.0–91.0]	65.3 ± 32.9 69.0 [35.0–90.0]	74.2 ± 27.7 78.0 [55.0–91.0]	0.171
CF liver disease	57.1%	54.8%	59.2%	0.678
Nasal polyps	36.3%	38.1%	34.7%	0.828
GERD	31.9%	40.5%	24.5%	0.119
Diabetes	20.9%	26.2%	16.3%	0.305
*P. aeruginosa* chronic or intermittent	75.8%	83.3%	69.4%	0.146

BMI—body mass index; FEV1%—forced expiratory volume in 1 s, GERD—gastroesophageal reflux disease.

**Table 3:** Daily doses of vitamins prior to the study. Mean ± SD is provided.

Vitamin	All Patients	Liposomal	Standard Vitamins	*p*
Vitamin A, RAE (retinol and β-carotene)	1638 ± 1816 1102 [550–2200]	1937 ± 2008 1102 [1100–2200]	1382 ± 1610 1100 [367–1837]	0.154
Vitamin D3, IU	3757 ± 2572 4000 [2000–5000]	4112 ± 2734 4000 [2000–5000]	3446 ± 2407 3500 [2000–5000]	0.226
Vitamin E, IU	264 ± 148 270 [135–400]	290 ± 169 300 [200–400]	241 ± 124 270 [135–350]	0.125
Vitamin K1, mg average daily	2.94 ± 2.50 2.86 [0.2–5.0]	3.16 ± 2.64 2.6 [1.4–5.0]	2.75 ± 2.39 2.86 [0.2–5.0]	0.445
Vitamin K2, µg (MK-7)	44.5 ± 76.4 0 [0–100]	36.9 ± 73.1 0 [0–0]	51.0 ± 79.4 0 [0–100]	0.380

**Table 4:** Changes in fat-soluble vitamin concentration and their levels in pancreatic-insufficient CF patients before and after supplementation with the novel liposomal formulation and the standard forms (control). Mean ± SD are given, along with two-sided *p* values from the unequal variances t test (statistically significant differences are presented in bold). Please note that the percentage of undercarboxylated osteocalcin reflects the functional insufficiency of vitamin K, hence lower concentrations and negative changes should be interpreted as beneficial.

Biomarker	Liposomal, *n* = 42	Standard Vitamins, *n* = 49	*p*
Start: all-*trans*-retinol, ng/mL	368.5 ± 101.3 351.5 [306.0–414.0]	366.1 ± 148.8 350.0 [277.0–443.0]	0.929
**End:** **all-*trans*-retinol, ng/mL**	**370.0 ± 116.5** **362.0 [293.0–451.0]**	**323.1 ± 100.6** **301.0 [268.0–410.0]**	**0.045**
Change: all-*trans*-retinol, ng/mL	1.48 ± 95.9 12.0 [−70.0–60.0]	−43.1 ± 121.4 −15.0 [−78.0–23.0]	0.054
Start: 25-OHD3, ng/mL	33.5 ± 15.8 31.2 [24.7–40.8]	30.7 ± 11.8 30.1 [21.4–38.3]	0.343
**End:** **25-OHD3, ng/mL**	**43.2 ± 16.6** **43.0 [35.3**–**56.3]**	**32.7 ± 11.5** **33.8 [26.7**–**40.2]**	**<0.001**
**Change:** **25-OHD3, ng/mL**	**9.7 ± 13.4** **10.5 [** **−1.3–19.5]**	**2.0 ± 9.8** **2.8 [** **−2.4–8.9]**	**0.004**
Start: α-tocoferol, µg/mL	7.5 ± 3.0 7.8 [4.8–9.0]	7.9 ± 2.8 7.8 [5.6–10.0]	0.540
**End: ** **α-tocoferol, µg/mL**	**9.0 ± 3.1** **8.9 [6.9–10.3]**	**7.7 ± 3.0** **7.1 [5.3–10.5]**	**0.037**
**Change: ** **α-tocoferol, µg/mL**	**1.5 ± 2.5** **1.9 [0.5–2.7]**	**−0.2 ± 1.6** **−0.1 [-0.9–0.9]**	**<0.001**
Start: %ucOC	30.2 ± 26.7 19.2 [9.6–5.28]	31.0 ± 27.4 23.3 [9.7–42.1]	0.878
**End:** **%ucOC**	**13.0 ± 11.2** **9.5 [5.6–15.0]**	**22.7 ± 22.0** **13.0 [8.5–30.4]**	**0.008**
Change: %ucOC	−17.2 ± 24.8 −4.0 [−40.3–(−0.3)]	−8.3 ± 18.5 −5.4 [−12.9–2.0]	0.060

%ucOC—percentage of undercarboxylated osteocalcin, 25-OHD3—25-hydroxyvitamin D3.

**Table 5:** The frequencies of vitamin A, D, E, and K deficiency at the start and → at the end of the trial. The changes in the frequency of insufficiency were compared using the Mann-Whitney U test with the continuity correction (statistically significant differences are presented in bold). The final frequencies of deficiencies were compared using the Fisher’s exact test.

Parameter	Liposomal	Control	P for the Prevalence Change	*p* for Final Prevalence
Vitamin A deficiency	23.8% → 28.6%	38.8% → 44.9%	0.927	0.131
Vitamin D deficiency	11.9% → 11.9%	22.4% → 8.2%	0.107	0.728
Vitamin E deficiency	28.6% → 7.1%	18.4% → 18.4%	0.255	0.134
**Vitamin K deficiency**	47.6% → **16.7%**	53.1% → **36.7%**	0.229	**0.037**

## Data Availability

The data may be available at a reasonable request.
